# Synovial Calprotectin in Suspected Periprosthetic Joint Infection After Total Knee Arthroplasty: Diagnostic Accuracy and Exploratory Adjunctive Value to Preoperative ICM Classification

**DOI:** 10.3390/antibiotics15080775

**Published:** 2026-08-12

**Authors:** Pavlos Altsitzioglou, Panayiotis Gavriil, Stavros Goumenos, Vasileios Karampikas, Anastasios Roustemis, Olga Savvidou, Panayiotis Papagelopoulos, Vasileios Kontogeorgakos

**Affiliations:** 1 First Department of Orthopaedics, ATTIKON University General Hospital, National and Kapodistrian University of Athens, 12462 Athens, Greece; pavlosaltsi@gmail.com (P.A.); vas.karampikas@gmail.com (V.K.); roustemisan@med.uoa.gr (A.R.); olgasavvidou@gmail.com (O.S.); pjportho@med.uoa.gr (P.P.); vkonto@med.uoa.gr (V.K.); 2 Leeds Major Trauma Centre, Leeds Teaching Hospitals NHS Trust, Leeds LS1 3EX, UK; stgoumenos@gmail.com

**Keywords:** periprosthetic joint infection, total knee arthroplasty, calprotectin, synovial biomarker, International Consensus Meeting, diagnostic accuracy, sonication, antibiotic exposure

## Abstract

Background/Objectives: Diagnosis of periprosthetic joint infection (PJI) after total knee arthroplasty remains challenging when standard criteria are inconclusive. This study evaluated the diagnostic accuracy of synovial calprotectin and its prespecified exploratory adjunctive value to preoperative 2018 International Consensus Meeting (ICM) classification. Methods: This prospective single-center diagnostic accuracy study included 35 consecutive patients with primary or revision total knee arthroplasty who underwent synovial calprotectin testing followed by surgery for suspected PJI. Calprotectin was measured using a lateral flow assay with a prespecified threshold of ≥50 mg/L. Diagnostic performance was assessed against a multidisciplinary composite postoperative reference standard that did not incorporate calprotectin. Results: Twenty-one patients were classified as infected and 14 as non-infected. Calprotectin yielded 18 true-positive, 11 true-negative, 3 false-positive, and 3 false-negative results, corresponding to 85.7% sensitivity, 78.6% specificity, 85.7% positive predictive value, 78.6% negative predictive value, and an area under the curve of 0.83. Preoperative ICM correctly classified 26 patients, misclassified one, and left eight inconclusive. Among the inconclusive cases, calprotectin correctly classified all six non-infected patients but neither of the two infected patients. The combined strategy classified all 35 patients, correctly classifying 32 and misclassifying three. Among 10 antibiotic-exposed patients—seven infected and three non-infected—no misclassifications occurred; this finding was descriptive. Conclusions: Synovial calprotectin showed good overall diagnostic performance in this surgically managed cohort. When applied to preoperative ICM-inconclusive cases, it increased classification yield but missed both infected cases and should not be used alone to exclude PJI. Larger blinded multicenter studies are required before routine implementation.

## 1. Introduction

Periprosthetic joint infection (PJI) remains one of the most serious complications after total knee arthroplasty (TKA), despite its relatively low incidence. Population-based and registry-linked studies have reported PJI rates after primary TKA of approximately 0.5–2%, while also indicating that registry estimates based only on revision for infection may underestimate the true incidence of clinically relevant PJI [1,2,3]. Despite this relatively low incidence, knee PJI carries substantial clinical consequences, as it is associated with repeated revision surgery, prolonged antimicrobial treatment, substantial reinfection risk, increased related financial burden, and higher morbidity and mortality compared with aseptic revision TKA [4,5,6,7,8]. Accurate early diagnosis is therefore essential, because differentiating septic from aseptic failure directly guides surgical and antimicrobial management, while missed or unexpectedly diagnosed infection at revision may compromise treatment strategy and outcome [9,10,11].

The 2018 International Consensus Meeting (ICM) criteria provide a widely accepted, evidence-based framework for PJI diagnosis by integrating major criteria with weighted serum, synovial, and intraoperative findings into a structured diagnostic score [12,13]. However, PJI diagnosis remains clinically challenging because no single universally accepted gold standard exists, and septic and aseptic failure may present with overlapping findings [9,10,11]. Importantly, a subset of cases remains inconclusive, particularly when diagnostic findings are borderline or discordant [14].

Conventional serum markers such as CRP and ESR remain practical first-line tests in suspected PJI, but their diagnostic performance is imperfect and they reflect systemic inflammation rather than local synovial infection biology [15,16]. Synovial WBC count and PMN% provide more direct assessment of intra-articular inflammation and remain central, accessible diagnostic anchors, yet their interpretation may be influenced by prior antibiotics, host comorbidities, implant-related debris, inflammatory conditions, and infection chronicity [15,17]. More advanced synovial biomarkers, including α-defensin and IL-6, have demonstrated high diagnostic accuracy and may complement conventional tests, but their routine use can be constrained by laboratory platform requirements, local availability, and differences between testing platforms [15,16,18,19]. These limitations support the need for practical adjunctive synovial biomarkers, particularly in cases with borderline or inconclusive findings.

Synovial calprotectin has emerged as a candidate synovial biomarker for PJI because it is released predominantly by neutrophils and monocytes at sites of local inflammation and therefore reflects local neutrophil-driven inflammation [20,21,22,23]. Early clinical studies described calprotectin as an inexpensive synovial marker with particular value for excluding chronic PJI, commonly using a 50 mg/L threshold [24]. Subsequent comparative studies and meta-analyses have reported high diagnostic accuracy for synovial calprotectin and have shown that rapid lateral-flow testing performs comparably to ELISA-based measurement, supporting its feasibility as a point-of-care test in routine arthroplasty practice [20,25].

Despite this growing evidence, calprotectin has been evaluated mainly as a standalone diagnostic test rather than as an adjunct to structured ICM-based classification. Whether calprotectin can reduce the unresolved fraction of ICM-inconclusive conventional knee PJI work-ups remains clinically relevant. The unresolved question is therefore not simply whether calprotectin is diagnostically accurate, but whether it provides incremental information when established preoperative findings remain inconclusive. Accordingly, the primary objective of this study was to evaluate the diagnostic accuracy of synovial calprotectin in patients with primary or revision TKA who underwent surgery for suspected PJI, permitting multidisciplinary postoperative reference-standard assessment. Secondary objectives were to assess agreement with preoperative 2018 ICM classification, evaluate the prespecified exploratory adjunctive value of calprotectin in ICM-inconclusive cases, and perform an exploratory analysis according to pre-aspiration antibiotic exposure.

## 2. Results

### 2.1. Cohort Characteristics

Participant flow is presented in Supplementary Figure S1. Of 82 patients evaluated for suspected PJI, three had no evaluable synovial aspirate and five were excluded because aspiration was performed within the first postoperative month. Seventy-four patients underwent evaluable preoperative calprotectin testing followed by surgery. Sixteen were excluded because follow-up was shorter than 1 year. Among the remaining 58 patients, 23 had a reconstruction type or anatomical site outside the scope of the present TKA analysis, leaving 35 patients with primary or revision TKA in the final cohort.

According to the multidisciplinary composite postoperative reference standard, 21 patients (60.0%) were classified as infected and 14 (40.0%) as non-infected. Among the infected cases, 17 (81.0%) had an acute clinical presentation and 4 (19.0%) had a chronic presentation. Baseline demographic, clinical, and laboratory characteristics are presented in Table 1, stratified by final infection status and pre-aspiration antibiotic exposure. No patient in the final TKA cohort was excluded because of missing diagnostic information or inability to establish the postoperative reference-standard classification.

Continuous variables are presented as median (interquartile range). Categorical variables are presented as *n* (%). Pre-aspiration antibiotic exposure was defined as systemic antibiotic administration within the 2 weeks preceding diagnostic aspiration. Postoperative timing was calculated from the interval between the most recent operation on the affected knee and diagnostic aspiration. Revision TKA construct in situ refers to the implant present at the diagnostic episode, not the operation performed after aspiration. CRP: C-reactive protein; ESR: erythrocyte sedimentation rate; WBC: white blood cell count; PMN: polymorphonuclear neutrophils; TKA: total knee arthroplasty.

### 2.2. Diagnostic Performance of Synovial Calprotectin

Using the prespecified threshold of ≥50 mg/L, synovial calprotectin correctly identified 18 of 21 infected patients and 11 of 14 non-infected patients. This corresponded to 18 true-positive, 11 true-negative, three false-positive, and three false-negative results. Case-level characteristics of the discordant calprotectin results are presented in Supplementary Table S1.

Overall sensitivity was 85.7% (95% CI, 65.4–95.0), specificity was 78.6% (95% CI, 52.4–92.4), positive predictive value was 85.7% (95% CI, 65.4–95.0), and negative predictive value was 78.6% (95% CI, 52.4–92.4). The positive likelihood ratio was 4.00 (95% CI, 1.45–11.07), and the negative likelihood ratio was 0.18 (95% CI, 0.06–0.54).

**Figure 1:**
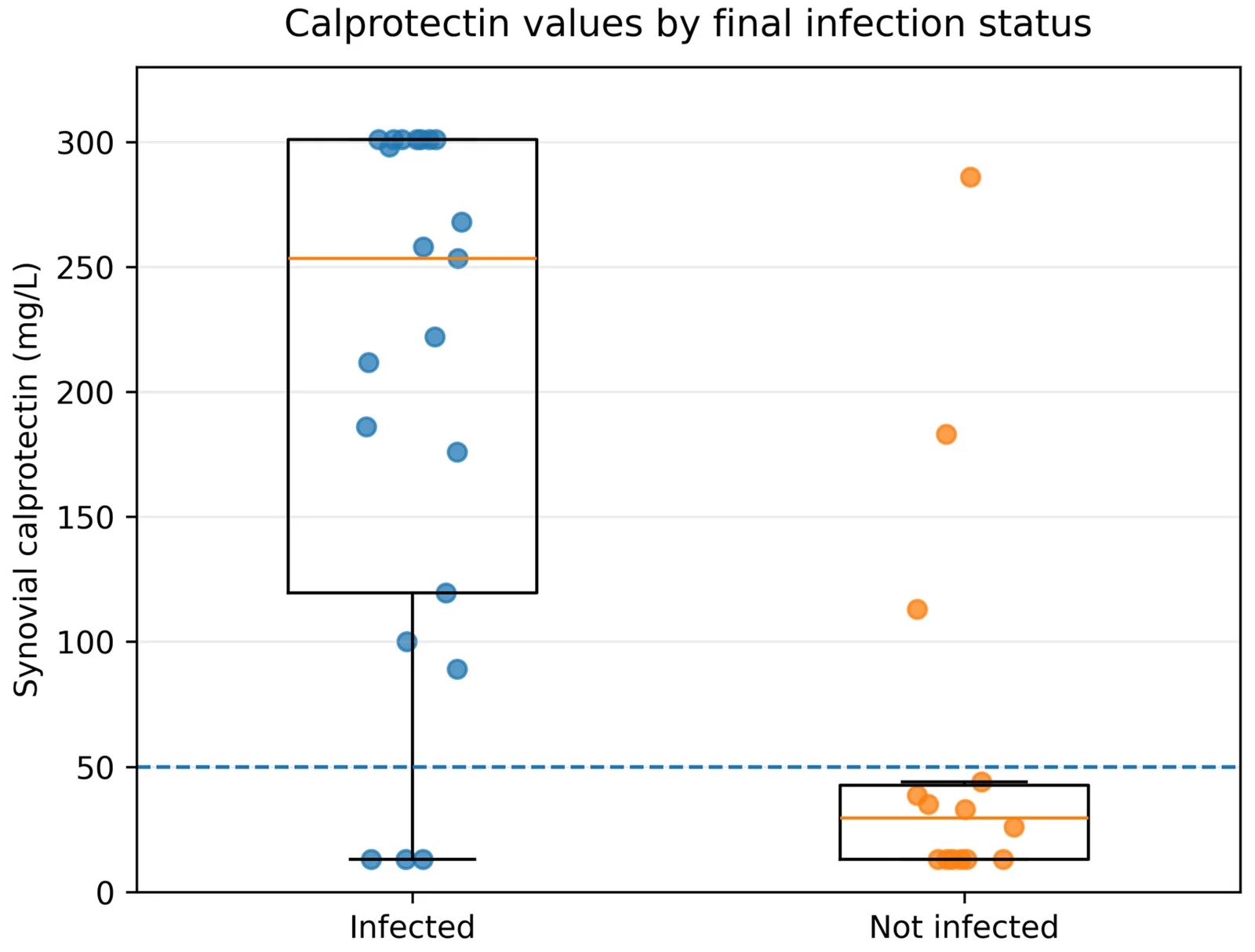
Distribution of synovial calprotectin values according to postoperative infection status in patients with primary or revision TKA. Individual calprotectin values are shown for infected and non-infected patients according to the multidisciplinary composite postoperative reference standard. The dashed horizontal line represents the prespecified positivity threshold of ≥50 mg/L. Values plotted at 13 and 301 mg/L represent boundary codes for assay results reported as <14 and >300 mg/L, respectively.

When analyzed as a continuous variable, synovial calprotectin demonstrated an AUC of 0.83 (95% CI, 0.68–0.96). The corresponding receiver operating characteristic curve, including the prespecified ≥50 mg/L operating point, is presented in Supplementary Figure S2. Median calprotectin was 253.4 mg/L (IQR, 119.6–301.0; range, 13.0–301.0) in infected patients and 29.5 mg/L (IQR, 13.0–42.7; range, 13.0–286.0) in non-infected patients. Values of 13 and 301 mg/L represent boundary codes for results reported by the assay as <14 and >300 mg/L, respectively. The distribution of calprotectin values according to postoperative infection status is shown in Figure 1.

In the exploratory antibiotic exposure analysis, 10 patients were antibiotic-exposed, including seven infected and three non-infected cases, whereas 25 were antibiotic-unexposed, including 14 infected and 11 non-infected cases. No calprotectin misclassifications occurred among antibiotic-exposed patients; sensitivity was 100% (95% CI, 64.6–100) and specificity was 100% (95% CI, 43.8–100). Among antibiotic-unexposed patients, sensitivity was 78.6% (95% CI, 52.4–92.4) and specificity was 72.7% (95% CI, 43.4–90.3). These estimates were compared descriptively without formal between-group hypothesis testing because of the small and unbalanced subgroup sizes and the non-randomized nature of antibiotic exposure. Complete threshold-based diagnostic estimates are presented in Table 2; subgroup-specific AUCs were not reported because the subgroup sizes did not permit reliable ROC estimation.

### 2.3. Preoperative ICM Classification and Adjunctive Value of Calprotectin

To evaluate calprotectin as an adjunct to preoperative ICM-based diagnosis, we examined the diagnostic yield of the preoperative 2018 ICM classification against the multidisciplinary composite postoperative reference standard. Among the 35 TKA cases, 20 were classified as infected, seven as aseptic, and eight as inconclusive according to preoperative ICM criteria. Thus, preoperative ICM provided a definitive classification in 27 cases (77.1%), while 8 cases (22.9%) remained inconclusive.

Of the 20 patients classified as infected by preoperative ICM, 19 were infected and one was non-infected according to the postoperative reference standard. All seven patients classified as aseptic by preoperative ICM were non-infected. Among the eight ICM-inconclusive cases, two were infected and six were non-infected. Preoperative ICM therefore correctly classified 26 of the 27 definitively classified cases (96.3%). Across the full cohort, it provided a correct definitive classification in 26 patients (74.3%), incorrectly classified one patient (2.9%), and left eight patients unresolved (22.9%).

Among the 27 cases with definitive preoperative ICM status, calprotectin agreed with ICM classification in 24 cases (88.9%): 19 of 20 ICM-infected cases were calprotectin-positive and five of seven ICM-aseptic cases were calprotectin-negative. Cohen’s κ was 0.70, and McNemar’s exact test showed no directional disagreement (*p* = 1.00). Agreement metrics were calculated only among the 27 definitively classified cases; ICM-inconclusive cases were analyzed separately.

Calprotectin alone, using the prespecified ≥50 mg/L threshold, classified all 35 patients and correctly classified 29 cases (82.9%), with 85.7% sensitivity and 78.6% specificity. All eight preoperative ICM-inconclusive cases had calprotectin values below 50 mg/L. Calprotectin therefore correctly classified all six non-infected inconclusive cases but misclassified both infected cases, yielding six of eight correct classifications overall. Case-level characteristics of the ICM-inconclusive cases are presented in Supplementary Table S2. Given the small subgroup and the two false-negative results, a negative calprotectin result could not exclude PJI in ICM-inconclusive cases.

In the prespecified exploratory adjunctive strategy, definitive preoperative ICM classifications were retained and calprotectin was applied only to ICM-inconclusive cases. This strategy assigned a binary classification to all 35 patients, correctly classifying 32 (91.4%; 95% CI, 77.6–97.0) and incorrectly classifying three (8.6%). The combined strategy yielded 19 true-positive, 13 true-negative, 1 false-positive, and two false-negative classifications. Sensitivity was 90.5% (95% CI, 71.1–97.3), specificity was 92.9% (95% CI, 68.5–98.7), PPV was 95.0% (95% CI, 76.4–99.1), and NPV was 86.7% (95% CI, 62.1–96.3).

**Figure 2:**
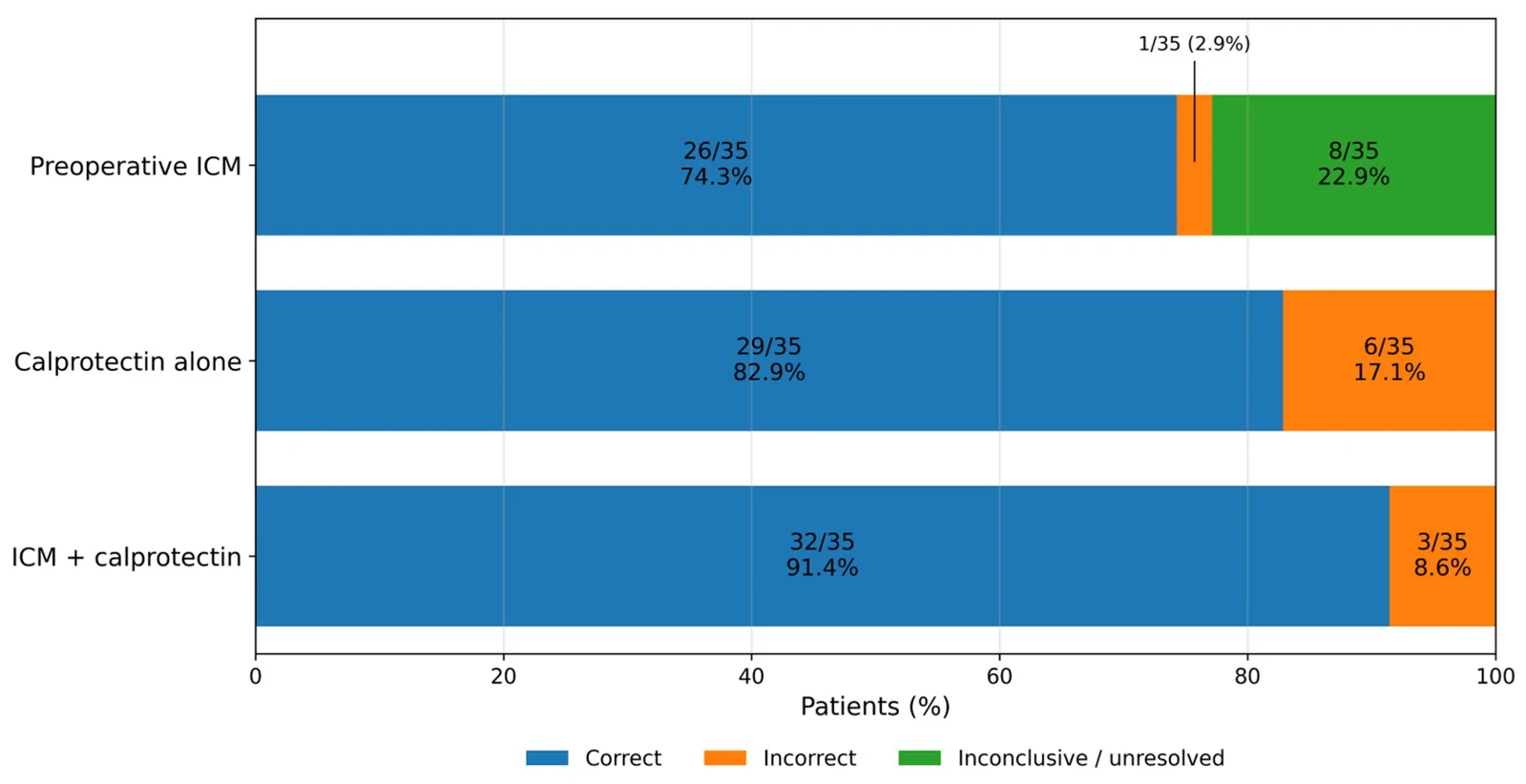
Full-cohort diagnostic classification yield of preoperative ICM alone, synovial calprotectin alone, and the prespecified exploratory ICM + calprotectin strategy. Values represent patient numbers and percentages of correct, incorrect, and unresolved classifications. Preoperative ICM correctly classified 26 patients, incorrectly classified 1, and left 8 unresolved. Calprotectin alone correctly classified 29 and incorrectly classified 6. The combined strategy correctly classified 32 and incorrectly classified 3, with no unresolved classifications.

Compared with preoperative ICM alone, the combined strategy increased the number of correct classifications from 26 to 32 and eliminated the eight unresolved classifications; however, the number of incorrect classifications increased from one to three because both infected ICM-inconclusive cases were classified as non-infected. Its principal effect was therefore increased classification yield rather than a demonstrated improvement in diagnostic safety. Full-cohort classification outcomes are summarized in Figure 2 and Table 3.

### 2.4. Microbiological Profile

Among the 21 patients classified as infected according to the multidisciplinary composite postoperative reference standard, preoperative aspiration cultures were positive in 11 cases (52.4%), while intraoperative tissue and/or sonication cultures yielded an organism in 20 cases (95.2%). Staphylococcal species predominated: Staphylococcus aureus was identified in six infected cases (28.6%), and coagulase-negative staphylococci or unspecified Staphylococcus spp. were identified in 10 cases (47.6%). Gram-negative organisms were identified in two cases (9.5%), and anaerobic or other low-virulence Gram-positive organisms in three cases (14.3%).

One infection was polymicrobial (4.8%), and one was culture-negative (4.8%). Overall, 22 organisms were recovered from 20 culture-positive infections: 19 infections were monomicrobial, while the polymicrobial infection yielded *Staphylococcus hominis*, *Gemella morbillorum*, and *Cutibacterium acnes*. The microbiological profile is summarized in Table 4.

## 3. Discussion

In this prospective single-center cohort of surgically managed patients with suspected PJI after primary or revision TKA, synovial calprotectin showed good overall diagnostic performance. Applying calprotectin to preoperative ICM-inconclusive cases increased classification yield and full-cohort correct classification, but both infected patients in this subgroup were missed. The antibiotic exposure analysis was exploratory and interpreted descriptively.

At the prespecified ≥50 mg/L threshold, calprotectin showed 85.7% sensitivity, 78.6% specificity, and an AUC of 0.83, although these estimates were lower than those reported in several previous studies and meta-analyses [19,20,21,24,25]. This difference may reflect the complex case spectrum of a surgically managed tertiary referral cohort and the use of a multidisciplinary postoperative reference standard rather than ICM, MSIS, or EBJIS criteria alone [15,19,20]. Early postoperative timing and non-infectious inflammatory or mechanical processes, including metallosis/wear, may also affect calprotectin values [26,27,28]. Case-level review identified no single explanation for discordant results. Two of the three false-positive cases were evaluated during the early postoperative period, and all three had sterile aspiration and intraoperative and/or sonication cultures. All three false-negative cases had sterile aspiration cultures, with organisms recovered only from intraoperative and/or sonication samples. These observations are descriptive and do not establish causality; detailed patient-level data are presented in Supplementary Table S1.

The main potential value of calprotectin was its use within, rather than instead of, a preoperative ICM-based workflow. The 2018 ICM criteria remain a structured and validated framework for PJI diagnosis, but some cases remain inconclusive when findings are borderline or discordant [12,13,14]. In our cohort, preoperative ICM correctly classified 26 patients, incorrectly classified one, and left eight unresolved. Applying calprotectin only to the inconclusive cases increased the number of correct classifications to 32 of 35 and removed the unresolved category; however, incorrect classifications increased from one to three because both infected inconclusive cases were classified as non-infected. All six non-infected inconclusive cases were correctly classified, whereas neither infected case was detected. The strategy therefore increased classification yield but did not safely resolve diagnostic uncertainty and should not be interpreted as a rule-out approach. This prespecified exploratory concept is consistent with previous studies evaluating calprotectin or other biomarkers in difficult PJI work-ups, including our prospective study in megaprosthetic reconstructions, but requires external validation before clinical implementation [23,26,29,30,31].

Several serum, synovial, and tissue-based tests have diagnostic value in PJI, but their clinical roles differ according to whether they are used to exclude infection, support infection, or identify the causative organism [32,33]. Synovial WBC count, PMN%, α-defensin, leukocyte esterase, and synovial CRP have shown strong diagnostic performance [15,16,19,33]. However, routine use of advanced biomarkers may depend on local availability, laboratory infrastructure, cost, and assay platform. In our diagnostic pathway, α-defensin and IL-6 were not routinely available. Calprotectin may therefore have a practical role as a rapid synovial adjunct that complements conventional diagnostic tests, particularly where more specialized biomarker platforms are unavailable.

Although antimicrobial regimens and operative strategies were not analyzed as outcomes, reporting the organism profile provides relevant microbiological context for the postoperative reference standard. Staphylococcal species predominated, with *Staphylococcus aureus* and coagulase-negative or unspecified *Staphylococcus* spp. representing the largest organism groups, consistent with the established microbiology of hip and knee PJI [34,35,36]. Intraoperative tissue and/or sonication cultures yielded an organism in 20 of 21 infected cases, while one infection was culture-negative. Culture-negative PJI remains a recognized diagnostic problem, particularly after antimicrobial exposure or in low-grade biofilm-related infection [37]. Multiple intraoperative tissue cultures and sonication were incorporated as complementary components of the postoperative reference standard, consistent with established evidence that sonication can improve recovery of biofilm-associated organisms and detection of polymicrobial infection [38,39,40]. The incremental yield of sonication over tissue culture alone was not separately evaluated because this was not an objective of the present diagnostic accuracy study.

Pre-aspiration antibiotic exposure was evaluated exploratorily because antimicrobial administration may reduce culture yield and complicate PJI assessment [26,37]. Diagnostic estimates were numerically higher in the 10 antibiotic-exposed patients than in the 25 antibiotic-unexposed patients. However, antibiotic exposure was non-randomized, and exposed patients had higher median CRP, serum WBC, synovial WBC, and PMN%, suggesting more overt inflammatory presentations and potential indication bias. Prior antibiotics, host factors, implant characteristics, and clinical context may also influence synovial biomarker interpretation [15]. The small subgroup size and wide confidence intervals preclude any conclusion that calprotectin is unaffected by antibiotic exposure; these findings should be considered descriptive and hypothesis-generating.

Strengths of this study include its prospective design, consecutive recruitment, prespecified ≥50 mg/L threshold, and inclusion of patients who subsequently underwent surgery, permitting operative assessment, collection of at least six intraoperative tissue specimens, sonication, and minimum 1-year follow-up. Calprotectin was not incorporated into the postoperative classification algorithm, and its comparison with preoperative ICM and adjunctive assessment in ICM-inconclusive cases was included in the study protocol. Several limitations should be acknowledged. Restriction to surgically managed patients allowed a robust postoperative reference standard and reflects the intended use of calprotectin in patients with substantial suspicion of PJI rather than as a general screening test. However, this selected high-pretest-probability cohort, in which 60.0% were infected, may introduce spectrum bias; predictive values—and potentially sensitivity and specificity—may not generalize to lower-risk aspiration populations. The single-center design, modest sample size, and absence of an a priori sample size calculation further limit generalizability and precision. The multidisciplinary postoperative reference standard differs from established ICM, MSIS, and EBJIS definitions, which may affect comparisons with previous studies. The treating surgeon and retrospective senior adjudicator were not formally blinded to calprotectin, so review bias cannot be excluded; however, calprotectin was excluded from the classification algorithm, and treatment decisions were supported in all cases by contemporaneous infectious diseases assessment performed without access to the calprotectin result. Preoperative ICM classification was based on available components because α-defensin, leukocyte esterase, and synovial CRP were unavailable and D-dimer was obtained in only five patients. Finally, timing from the most recent operation was heterogeneous, and the antibiotic exposure and ICM-inconclusive subgroup analyses remain exploratory. Larger blinded multicenter studies are required to validate these findings.

## 4. Materials and Methods

### 4.1. Study Design and Participants

This was a prospective single-center diagnostic accuracy study evaluating synovial calprotectin for the diagnosis of periprosthetic joint infection (PJI). The study was conducted at a tertiary referral center for complex arthroplasty, orthopedic infection, and joint reconstruction. Between September 2023 and December 2025, consecutive adult patients with a non-pelvic joint reconstruction evaluated for suspected PJI were screened for inclusion.

For the present analysis, patients were eligible if they were ≥18 years old, had a primary or revision total knee arthroplasty (TKA) construct in situ, underwent diagnostic aspiration with synovial calprotectin testing for suspected PJI, and subsequently underwent surgery with operative and microbiological assessment within 14 days after aspiration. Minimum follow-up of 1 year after surgery was required.

Patients were excluded if no evaluable synovial aspirate was obtained, aspiration was performed within the first month after the most recent operation on the affected knee, essential diagnostic information was missing, no definitive postoperative reference-standard classification could be established, follow-up was shorter than 1 year, or the patient had a megaprosthetic reconstruction or an arthroplasty at another anatomical site.

Postoperative timing was categorized according to the interval between the most recent operation on the affected knee and diagnostic aspiration as early postoperative (1 to <3 months), delayed (3 months to ≤2 years), or late (>2 years). Among infected cases, acute versus chronic presentation was recorded according to the contemporaneous clinical diagnosis.

The study was designed and reported according to STARD principles.

### 4.2. Index Test: Synovial Calprotectin

Synovial calprotectin was measured using the Lyfstone^®^ Calprotectin for Synovial Fluid lateral-flow assay (CALPRO AS, Lysaker, Norway) according to the manufacturer’s instructions. Briefly, 20 μL of synovial fluid was added to 2 mL of the supplied dilution buffer, and 80 μL of the diluted sample was applied to the test cassette. Results were measured after 15 min using the dedicated Lyfstone^®^ Reader smartphone application.

The device measurement range was 14–300 mg/L, the quantitative range was 24–300 mg/L, and the manufacturer-reported limit of detection was 2.99 mg/L. Results displayed as <14 mg/L or >300 mg/L were entered as 13 mg/L and 301 mg/L, respectively, for continuous analyses; these values represented boundary codes rather than exact concentrations outside the assay range. Bloody, viscous, or debris-containing aspirates were not excluded and were processed using the same manufacturer protocol. No invalid or uninterpretable tests occurred.

A prespecified threshold of ≥50 mg/L defined a positive result; values below 50 mg/L were considered negative. The threshold was specified before review of the study outcomes, was based on the manufacturer-defined high-risk threshold and previous studies using the same assay platform, and was not optimized using the present cohort [23,26]. At the time of testing, operators were aware that patients were being evaluated for suspected PJI but were unaware of the subsequent operative microbiology and final postoperative reference-standard classification.

### 4.3. Multidisciplinary Postoperative Reference Standard

The postoperative reference standard was a multidisciplinary composite classification based on conventional clinical, operative, microbiological, sonication, and minimum 1-year follow-up data. Synovial calprotectin was not incorporated into the classification algorithm.

During clinical care, the treating surgeon established the working diagnosis and treatment plan with contemporaneous input from the hospital infectious diseases service in all cases. The infectious diseases specialists did not have access to the synovial calprotectin result, were not involved in the study protocol or analysis, and their assessment guided the decision to proceed with revision surgery and antimicrobial management. After completion of follow-up, the study’s senior principal investigator retrospectively reviewed the conventional clinical, operative, microbiological, sonication, and follow-up information and confirmed the treating surgeon’s classification in all cases. No disagreements occurred. The treating surgeon and senior principal investigator were not formally blinded to calprotectin; however, calprotectin remained exploratory, was not used as a classification criterion, and did not determine operative or antimicrobial treatment selection.

Classification followed a hierarchical approach. Infection was confirmed by a sinus tract or fistula communicating with the prosthesis or by at least two separate microbiological specimens yielding the same organism. In the absence of these confirmatory findings, infection required concordance among the clinical course, macroscopic operative findings, conventional serum and synovial inflammatory markers, aspiration and intraoperative cultures, sonication or prolonged culture results when applicable, and follow-up.

A single positive culture, including isolation of a low-virulence organism, was not considered diagnostic in isolation. Such cases were classified as infected only when supported by concordant clinical or operative findings, conventional inflammatory marker abnormalities not otherwise explained, additional microbiological or sonication findings, and the subsequent clinical course. Culture-negative PJI required compelling clinical or operative evidence, including persistent wound drainage or dehiscence, a communicating sinus tract or fistula, purulence, or other macroscopic evidence of infection, together with supportive conventional laboratory and follow-up findings.

Patients were classified as non-infected when clinical, operative, microbiological, and sonication findings did not support infection and no evidence of PJI developed during a minimum 1-year follow-up. Cases with conflicting or insufficient evidence to establish either classification were considered inconclusive and excluded from the diagnostic accuracy analysis. No case in the final cohort remained inconclusive under the postoperative reference standard.

### 4.4. Microbiological Assessment

Preoperative aspiration cultures and intraoperative microbiological results were reviewed for all included patients. When sufficient synovial fluid was available, aspiration fluid was inoculated into aerobic and anaerobic blood culture bottles according to institutional protocol.

During surgery, at least six separate deep periprosthetic tissue specimens were obtained in every case and submitted individually for microbiological culture. Removed implants or polyethylene components were processed with sonication, and the resulting sonication fluid was cultured as part of the microbiological work-up. Standard aerobic and anaerobic cultures were performed, with prolonged incubation when slow-growing or low-virulence organisms were clinically suspected. Organism identification and antimicrobial susceptibility testing were performed by the hospital microbiology laboratory using standard clinical procedures.

For descriptive analysis, organisms were grouped as *Staphylococcus aureus*, coagulase-negative staphylococci or unspecified *Staphylococcus* spp., Gram-negative organisms, and anaerobic or other low-virulence Gram-positive organisms. Polymicrobial infection was defined as isolation of more than one clinically significant organism from the same infection episode. Culture-negative infection was defined as infection according to the postoperative reference standard without pathogen recovery from aspiration, intraoperative tissue, or sonication cultures. Operative and antimicrobial management was individualized by the multidisciplinary treating team and was not analyzed because treatment selection and treatment outcomes were outside the prespecified diagnostic accuracy objectives.

### 4.5. Preoperative ICM Classification and Diagnostic Strategies

The preoperative 2018 International Consensus Meeting (ICM) classification was determined using only information available before surgery. A sinus tract communicating with the prosthesis or two concordant preoperative cultures constituted a major criterion and classified the patient as infected. In the absence of a major criterion, the minor-criteria score was calculated according to the published 2018 ICM framework. A score of ≥6 was classified as infected, 2–5 as inconclusive, and 0–1 as aseptic [13]. α-Defensin, leukocyte esterase, and synovial CRP were not available in this diagnostic pathway. D-dimer was included when available. Missing components were not imputed and contributed no points. Three prespecified diagnostic strategies were evaluated against the postoperative reference standard: 1. Preoperative ICM alone—ICM-infected and ICM-aseptic cases were considered definitively classified, whereas ICM-inconclusive cases were retained as unresolved. 2. Calprotectin alone—all patients were classified as positive or negative using the prespecified ≥50 mg/L threshold. 3. Preoperative ICM + calprotectin—definitive preoperative ICM classifications were retained, and the prespecified ≥50 mg/L calprotectin threshold was applied only to ICM-inconclusive cases.

Comparison with preoperative ICM and assessment of calprotectin’s adjunctive value in ICM-inconclusive cases were included in the study protocol. The combined strategy was prespecified but exploratory because of the expected small number of ICM-inconclusive cases.

### 4.6. Outcomes and Statistical Analysis

The primary outcome was the diagnostic accuracy of synovial calprotectin for detecting PJI against the multidisciplinary postoperative reference standard. Secondary outcomes included agreement between calprotectin and definitive preoperative ICM classification, the full-cohort classification yield of preoperative ICM, calprotectin performance in ICM-inconclusive cases, diagnostic performance of the prespecified exploratory ICM + calprotectin strategy, and an exploratory analysis according to pre-aspiration antibiotic exposure. Pre-aspiration antibiotic exposure was defined as systemic antibiotic administration within the 2 weeks preceding diagnostic aspiration.

Continuous variables were summarized as median and interquartile range, and categorical variables as counts and percentages. Baseline characteristics were presented descriptively without formal between-group hypothesis testing. No a priori sample size calculation was performed because this was an exploratory diagnostic accuracy study based on consecutive eligible cases.

True-positive, true-negative, false-positive, and false-negative results were tabulated. Sensitivity, specificity, positive predictive value, negative predictive value, positive likelihood ratio, negative likelihood ratio, and overall correct classification were calculated using the prespecified ≥50 mg/L threshold. Ninety-five percent confidence intervals for sensitivity, specificity, predictive values, and correct classification proportions were calculated using the Wilson method. Confidence intervals for likelihood ratios were estimated on the log scale.

The area under the receiver operating characteristic curve (AUC) was calculated using continuous calprotectin values for the full cohort. Its 95% confidence interval was estimated using 10,000 nonparametric patient-level bootstrap resamples drawn with replacement. The AUC was recalculated in each resample; resamples containing only one reference standard category were excluded, and the 2.5th and 97.5th percentiles of the bootstrap distribution were reported. Subgroup-specific AUCs were not reported because the small antibiotic exposure subgroups did not permit reliable ROC estimation.

Agreement between calprotectin and preoperative ICM classification was assessed using Cohen’s κ among the 27 patients with definitive preoperative ICM status. ICM-inconclusive cases were excluded from the agreement analysis and evaluated separately. Directional disagreement was assessed using McNemar’s exact test. For full-cohort diagnostic strategy comparisons, the numbers and percentages of correct, incorrect, and unresolved classifications were reported. Preoperative ICM-inconclusive cases were retained as unresolved rather than excluded or assigned to an artificial binary category. The combined strategy provided a binary classification, for which true-positive, true-negative, false-positive, and false-negative results were also reported.

Diagnostic metrics were calculated separately for antibiotic-exposed and antibiotic-unexposed patients. These analyses were descriptive, with no formal between-group hypothesis testing, because antibiotic exposure was non-randomized and the subgroup sizes were small and unbalanced. No imputation was performed for missing data.

## 5. Conclusions

Synovial calprotectin demonstrated good overall diagnostic performance in surgically managed patients with suspected PJI after primary or revision TKA. In preoperative ICM-inconclusive cases, it increased classification yield but missed both infected cases; therefore, a negative result should not be used alone to exclude PJI in diagnostically uncertain patients. The combined strategy remains exploratory and requires larger, blinded, multicenter validation before incorporation into routine diagnostic algorithms.

## Figures and Tables

**Table 1:** Baseline characteristics of the conventional knee arthroplasty cohort (*n* = 35).

Panel A. Stratification by Final Infection Status					
Variable	Overall (*n* = 35)		Infected (*n* = 21)		Non-Infected (*n* = 14)
Age (years)	72.0 (65.0–76.0)		73.0 (69.0–78.0)		72.0 (65.0–73.0)
Male sex	13 (37.1%)		8 (38.1%)		5 (35.7%)
CRP (mg/L)	22.0 (5.0–70.4)		41.8 (30.0–116.0)		5.0 (3.3–11.3)
ESR (mm/h)	35.0 (25.0–45.0)		40.0 (35.0–55.0)		25.0 (15.0–33.8)
Serum WBC (×10^9^/L)	7.9 (6.2–9.3)		9.0 (7.5–12.0)		6.3 (5.2–7.5)
Synovial WBC (K/μL)	6.95 (2.75–17.00)		15.20 (6.95–40.00)		1.83 (0.74–4.05)
PMN (%)	85.0 (45.0–90.0)		90.0 (85.0–96.0)		35.0 (25.8–74.0)
Preoperative aspiration culture positive	11 (31.4%)		11 (52.4%)		0 (0.0%)
Pre-aspiration antibiotic exposure (≤14 days)	10 (28.6%)		7 (33.3%)		3 (21.4%)
Sinus tract present	1 (2.9%)		1 (4.8%)		0 (0.0%)
Time from most recent surgery to aspiration (years)	1.0 (0.3–1.4)		1.0 (0.2–1.3)		1.0 (0.6–1.4)
Postoperative timing category					
Early postoperative: 1 to <3 months	8 (22.9%)		6 (28.6%)		2 (14.3%)
Delayed: 3 months to ≤2 years	21 (60.0%)		12 (57.1%)		9 (64.3%)
Late: >2 years	6 (17.1%)		3 (14.3%)		3 (21.4%)
Inflammatory arthritis	0 (0.0%)		0 (0.0%)		0 (0.0%)
Metallosis/wear present	7 (20.0%)		5 (23.8%)		2 (14.3%)
Revision TKA construct in situ	6 (17.1%)		5 (23.8%)		1 (7.1%)
**Panel B. Stratification by pre-aspiration antibiotic exposure**					
**Variable**		**Antibiotic-exposed** **(*n* = 10)**		**Antibiotic-unexposed** **(*n* = 25)**	
Age (years)		73.0 (56.3–76.8)		72.0 (69.0–76.0)	
Male sex		6 (60.0%)		7 (28.0%)	
Final infection status: infected		7 (70.0%)		14 (56.0%)	
CRP (mg/L)		90.0 (39.3–149.8)		14.0 (5.0–37.0)	
ESR (mm/h)		36.5 (25.5–43.8)		35.0 (25.0–45.0)	
Serum WBC (×10^9^/L)		9.45 (8.48–12.23)		7.10 (6.00–8.60)	
Synovial WBC (K/μL)		14.00 (4.28–35.00)		6.35 (2.00–9.40)	
PMN (%)		92.5 (90.0–96.0)		77.8 (30.0–85.0)	
Preoperative aspiration culture positive		4 (40.0%)		7 (28.0%)	
Sinus tract present		0 (0.0%)		1 (4.0%)	
Time from most recent surgery to aspiration (years)		0.75 (0.28–1.00)		1.00 (0.30–1.80)	
Revision TKA construct in situ		1 (10.0%)		5 (20.0%)	

**Table 2:** Diagnostic performance of synovial calprotectin in suspected PJI after TKA.

Metric	Overall Cohort (*n* = 35)	Antibiotic-Exposed (*n* = 10)	Antibiotic-Unexposed (*n* = 25)
**Infected/non-infected**	21/14	7/3	14/11
**TP/TN/FP/FN**	18/11/3/3	7/3/0/0	11/8/3/3
**Sensitivity**	85.7% (65.4–95.0)	100% (64.6–100)	78.6% (52.4–92.4)
**Specificity**	78.6% (52.4–92.4)	100% (43.8–100)	72.7% (43.4–90.3)
**PPV**	85.7% (65.4–95.0)	100% (64.6–100)	78.6% (52.4–92.4)
**NPV**	78.6% (52.4–92.4)	100% (43.8–100)	72.7% (43.4–90.3)
**LR+**	4.00 (1.45–11.07)	∞	2.88 (1.06–7.86)
**LR−**	0.18 (0.06–0.54)	0.00	0.29 (0.10–0.86)
**AUC**	0.83 (0.68–0.96)	-	-

Diagnostic performance was calculated using the prespecified ≥50 mg/L threshold. Values are presented with 95% confidence intervals where applicable. Antibiotic exposure was defined as systemic antibiotic administration within 2 weeks before aspiration. Antibiotic exposure analyses were exploratory and interpreted descriptively; no formal between-group hypothesis testing was performed. Subgroup-specific AUCs were not reported because the small subgroup sizes did not permit reliable ROC estimation. TP: true positive; TN: true negative; FP: false positive; FN: false negative; PPV: positive predictive value; NPV: negative predictive value; LR: likelihood ratio; AUC: area under the receiver operating characteristic curve; ∞ indicates an infinite LR+ resulting from zero false-positive results (specificity = 100%).

**Table 3:** Full-cohort diagnostic outcome counts for preoperative ICM and the combined strategy.

Strategy	TP	TN	FP	FN	Unresolved (Infected/Non-infected)	Correct *n* (%)	Incorrect *n* (%)	Unresolved *n* (%)
**Preoperative ICM**	19	7	1	0	2/6	26 (74.3%)	1 (2.9%)	8 (22.9%)
**ICM + calprotectin**	19	13	1	2	0/0	32 (91.4%)	3 (8.6%)	0 (0.0%)

For preoperative ICM, TP, TN, FP, and FN refer to definitive infected or aseptic classifications; the ICM-inconclusive cases were retained as unresolved and are reported separately. The combined strategy retained definitive preoperative ICM classifications and applied the prespecified ≥50 mg/L calprotectin threshold only to ICM-inconclusive cases. TP: true positive; TN: true negative; FP: false positive; FN: false negative; ICM: International Consensus Meeting.

**Table 4:** Microbiological profile of infected conventional knee PJI cases (*n* = 21).

Microbiological Variable	*n* (%)
**Culture yield**	
Preoperative aspiration culture positive	11 (52.4%)
Intraoperative tissue and/or sonication culture positive	20 (95.2%)
Culture-negative infection	1 (4.8%)
Polymicrobial infection	1 (4.8%)
**Organism groups**	
*Staphylococcus aureus*	6 (28.6%)
Coagulase-negative staphylococci/unspecified *Staphylococcus* spp.	10 (47.6%)
Gram-negative organisms	2 (9.5%)
Anaerobic or other low-virulence Gram-positive organisms	3 (14.3%)
**Specific isolates**	
*Staphylococcus aureus*	6 (28.6%)
*Staphylococcus epidermidis*	4 (19.0%)
*Staphylococcus haemolyticus*	2 (9.5%)
*Staphylococcus hominis*	2 (9.5%)
Unspecified *Staphylococcus* spp.	2 (9.5%)
*Enterobacter cloacae*	1 (4.8%)
*Acinetobacter schindleri*	1 (4.8%)
*Cutibacterium acnes*	2 (9.5%)
*Gemella morbillorum*	1 (4.8%)
*Peptostreptococcus* spp.	1 (4.8%)

Values are presented as *n* (% of the 21 infected cases). Twenty-two organisms were recovered from 20 culture-positive infections: 19 infections were monomicrobial, and one polymicrobial infection yielded *Staphylococcus hominis*, *Gemella morbillorum*, and *Cutibacterium acnes*. One additional infected case was culture-negative. Organism-group and specific-isolate rows are not mutually exclusive, and percentages use infected cases rather than total isolates as the denominator. CoNS: coagulase-negative staphylococci.

## Data Availability

The data presented in this study are available from the corresponding author upon reasonable request. The data are not publicly available due to privacy and ethical restrictions, as they contain patient-level clinical information.

## Supplementary Materials

The following supporting information can be downloaded at: https://www.mdpi.com/article/10.3390/antibiotics15080775/s1, Figure S1: STARD-style participant flow diagram; Figure S2: Receiver operating characteristic curve of synovial calprotectin against the multidisciplinary composite postoperative reference standard; Table S1: Case-level characteristics of calprotectin-discordant cases; Table S2: Case-level characteristics of preoperative ICM-inconclusive TKA cases.
