# Supplementary material for: Synovial Calprotectin in Suspected Periprosthetic Joint Infection After Total Knee Arthroplasty: Diagnostic Accuracy and Exploratory Adjunctive Value to Preoperative ICM Classification
Source: Antibiotics (Basel). 2026 Aug 12;15(8):775. doi: 10.3390/antibiotics15080775 (PMC13509904; doi:10.3390/antibiotics15080775)
Supplement: Supplementary file 1 [file antibiotics-15-00775-s001.zip › Figure_S1_STARD_Participant_Flow.pdf]

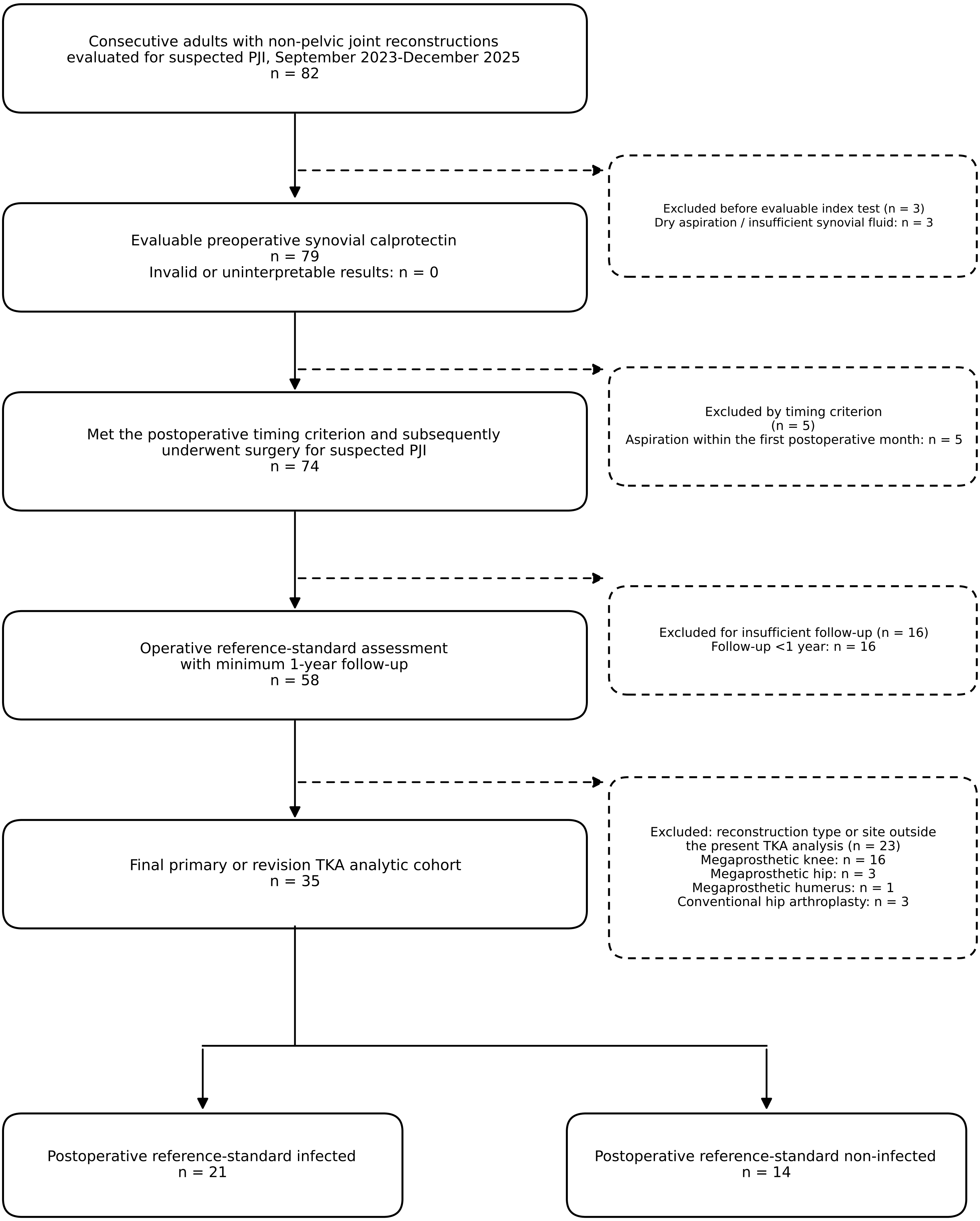
Consecutive adults with non-pelvic joint reconstructions evaluated for suspected PJI, September 2023-December 2025
n = 82
Excluded before evaluable index test (n = 3)
Dry aspiration / insufficient synovial fluid: n = 3
Evaluable preoperative synovial calprotectin
n = 79
Invalid or uninterpretable results: n = 0
Excluded by timing criterion (n = 5)
Aspiration within the first postoperative month: n = 5
Met the postoperative timing criterion and subsequently underwent surgery for suspected PJI
n = 74
Excluded for insufficient follow-up (n = 16)
Follow-up <1 year: n = 16
Operative reference-standard assessment with minimum 1-year follow-up
n = 58
Excluded: reconstruction type or site outside the present TKA analysis (n = 23)
Megaprosthetic knee: n = 16
Megaprosthetic hip: n = 3
Megaprosthetic humerus: n = 1
Conventional hip arthroplasty: n = 3
Final primary or revision TKA analytic cohort
n = 35
Postoperative reference-standard infected
n = 21
Postoperative reference-standard non-infected
n = 14
