# Supplementary material for: Synovial Calprotectin in Suspected Periprosthetic Joint Infection After Total Knee Arthroplasty: Diagnostic Accuracy and Exploratory Adjunctive Value to Preoperative ICM Classification
Source: Antibiotics (Basel). 2026 Aug 12;15(8):775. doi: 10.3390/antibiotics15080775 (PMC13509904; doi:10.3390/antibiotics15080775)
Supplement: Supplementary file 1 [file antibiotics-15-00775-s001.zip › Figure_S2_ROC_Calprotectin.pdf]

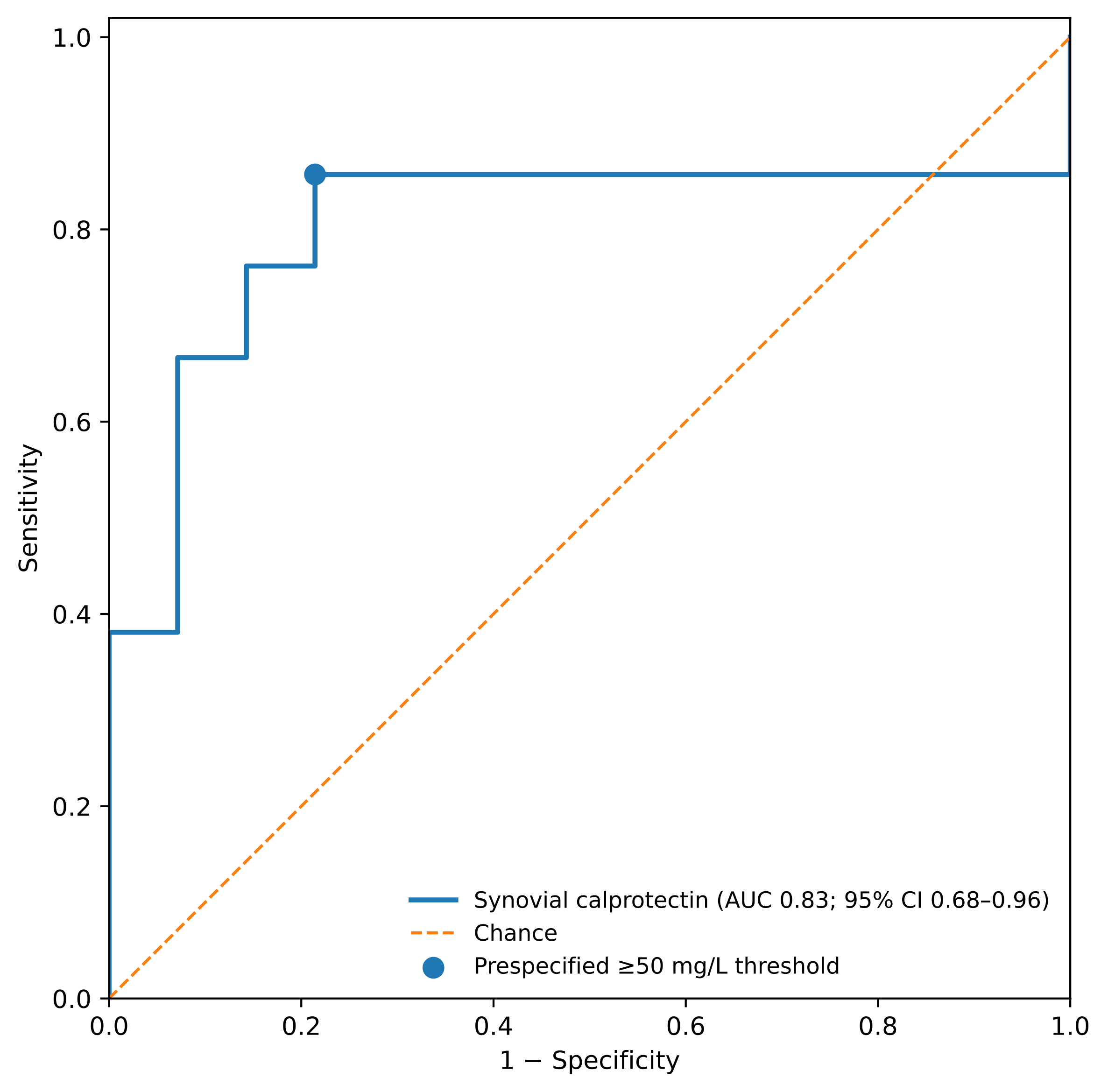

Synovial calprotectin (AUC 0.83; 95% CI 0.68–0.96)
Chance
Prespecified ≥50 mg/L threshold
Sensitivity
1 − Specificity
0.0
0.2
0.4
0.6
0.8
1.0
