# Supplementary material for: Synovial Calprotectin in Suspected Periprosthetic Joint Infection After Total Knee Arthroplasty: Diagnostic Accuracy and Exploratory Adjunctive Value to Preoperative ICM Classification
Source: Antibiotics (Basel). 2026 Aug 12;15(8):775. doi: 10.3390/antibiotics15080775 (PMC13509904; doi:10.3390/antibiotics15080775)
Supplement: Supplementary file 1 [file antibiotics-15-00775-s001.zip › Table_S1_Calprotectin_Discordant_Cases.pdf]

Supplementary Table S1. Case-level characteristics of calprotectin-discordant cases.

Case	Error type	Postoperative status	Preop. ICM	Calprotectin (mg/L)	CRP (mg/L)	ESR (mm/h)	Serum WBC (×10⁹/L)	Synovial WBC (K/μL)	PMN (%)	Aspiration culture	Intraoperative / sonication microbiology	Antibiotics ≤14 d	Time from most recent surgery	TKA construct	Metallosis / wear	Data-level context
D1	False positive	Non-infected	Aseptic	183.0 (Positive)	18.5	15	7.65	2.00	30	Sterile	Sterile	No	0.10 y (Early)	Primary	No	Sterile cultures; early postoperative inflammation was a possible contributor.
D2	False negative	Infected	Inconclusive	13.0 (Negative)	9.0	35	4.00	78.45	93	Sterile	Staphylococcus spp.	No	0.30 y (Delayed)	Revision	No	Sterile aspiration; organism recovered intraoperatively and/or after sonication; preoperative ICM inconclusive.
D3	False negative	Infected	Infected	13.0 (Negative)	154.0	40	6.80	9.40	85	Sterile	Cutibacterium acnes	No	1.20 y (Delayed)	Revision	Yes	Sterile aspiration; metallosis/wear present; infection established by the postoperative reference standard.
D4	False negative	Infected	Inconclusive	13.0 (Negative)	5.0	35	7.50	7.10	30	Sterile	Acinetobacter schindleri	No	0.20 y (Early)	Primary	Yes	Sterile aspiration; organism recovered intraoperatively and/or after sonication; preoperative ICM inconclusive.
D5	False positive	Non-infected	Infected	286.0 (Positive)	3.0	35	4.00	3.60	80	Sterile	Sterile	No	0.15 y (8 weeks; early)	Primary	No	Sterile cultures; early postoperative inflammation was a possible contributor.
D6	False positive	Non-infected	Aseptic	113.0 (Positive)	5.0	35	6.00	0.50	5	Sterile	Sterile	No	1.00 y (Delayed)	Primary	No	Sterile cultures with low synovial WBC/PMN; no clear data-level explanation.

Values are presented descriptively. Discordance was defined against the multidisciplinary composite postoperative reference standard using the prespecified ≥50 mg/L calprotectin threshold. Intraoperative tissue-culture and sonication findings were recorded jointly in the source database. Timing categories were early (1 to <3 months), delayed (3 months to ≤2 years), and late (>2 years). Data-level context is descriptive and should not be interpreted as causal attribution. ICM: International Consensus Meeting; WBC: white blood cell count; PMN: polymorphonuclear neutrophils; TKA: total knee arthroplasty.
