# Supplementary material for: Synovial Calprotectin in Suspected Periprosthetic Joint Infection After Total Knee Arthroplasty: Diagnostic Accuracy and Exploratory Adjunctive Value to Preoperative ICM Classification
Source: Antibiotics (Basel). 2026 Aug 12;15(8):775. doi: 10.3390/antibiotics15080775 (PMC13509904; doi:10.3390/antibiotics15080775)
Supplement: Supplementary file 1 [file antibiotics-15-00775-s001.zip › Table_S2_ICM_Inconclusive_Cases.pdf]

Supplementary Table S2. Case-level characteristics of preoperative ICM-inconclusive TKA cases.

Case	Postoperative reference status	Calprotectin (mg/L)	Calprotectin result	Correct by calprotectin	CRP (mg/L)	ESR (mm/h)	Synovial WBC (K/μL)	PMN (%)	Aspiration culture	Intraoperative / sonication microbiology	Antibiotics ≤14 d	Time from most recent surgery	TKA construct	Metallosis / wear
S2-1	Non-infected	13.0	Negative	Yes	18.0	30	6.95	77.8	Sterile	Sterile	No	5.00 y (Late)	Primary	Yes
S2-2	Infected	13.0	Negative	No	9.0	35	78.45	93.0	Sterile	Staphylococcus spp.	No	0.30 y (Delayed)	Revision	No
S2-3	Non-infected	13.0	Negative	Yes	161.0	55	0.19	90.0	Sterile	Sterile	Yes	1.00 y (Delayed)	Primary	No
S2-4	Non-infected	13.0	Negative	Yes	3.0	15	8.50	25.0	Sterile	Sterile	No	1.00 y (Delayed)	Primary	No
S2-5	Infected	13.0	Negative	No	5.0	35	7.10	30.0	Sterile	Acinetobacter schindleri	No	0.20 y (Early)	Primary	Yes
S2-6	Non-infected	13.0	Negative	Yes	4.15	25	5.64	40.0	Sterile	Sterile	No	3.00 y (Late)	Primary	No
S2-7	Non-infected	13.0	Negative	Yes	3.0	25	4.20	30.0	Sterile	Sterile	Yes	0.50 y (Delayed)	Primary	No
S2-8	Non-infected	33.0	Negative	Yes	12.0	20	1.00	28.0	Sterile	Sterile	No	1.00 y (Delayed)	Primary	No

All listed cases were classified as inconclusive by the preoperative 2018 ICM criteria. Calprotectin positivity was defined as ≥50 mg/L. All eight calprotectin results were negative: all six non-infected cases were correctly classified, whereas both infected cases were missed. Intraoperative tissue-culture and sonication findings were recorded jointly in the source database. Timing categories were early (1 to <3 months), delayed (3 months to ≤2 years), and late (>2 years). ICM: International Consensus Meeting; WBC: white blood cell count; PMN: polymorphonuclear neutrophils; TKA: total knee arthroplasty.
